# Indocyanine green-enhanced fluorescence to assess bowel perfusion during laparoscopic colorectal resection

**DOI:** 10.1007/s00464-015-4540-z

**Published:** 2015-10-20

**Authors:** Luigi Boni, Giulia David, Gianlorenzo Dionigi, Stefano Rausei, Elisa Cassinotti, Abe Fingerhut

**Affiliations:** Minimally Invasive Surgery Research Center, Department of Surgical and Morphological Sciences, University of Insubria, 21100 Varese, Italy; Section for Surgical Research, Department of Surgery, Medical University of Graz, Graz, Austria; First Department of Surgery, Hippokration University Hospital, University of Athens, Athens, Greece

**Keywords:** ICG, Indocyanine green, Bowel perfusion, Anastomotic leaks, Colorectal resection, Fluorescence

## Abstract

**Aims:**

Anastomotic leakage after colorectal surgery is a severe complication. One possible cause of anastomotic leakage is insufficient vascular supply. The aim of this study was to evaluate the feasibility and the usefulness of intraoperative assessment of vascular anastomotic perfusion in colorectal surgery using indocyanine green (ICG)-enhanced fluorescence.

**Methods:**

Between May 2013 and October 2014, all anastomosis and resection margins in colorectal surgery were investigated using fluorescence angiography (KARL STORZ GmbH & Co. KG, Tuttlingen, Germany) intraoperatively to assess colonic perfusion prior to and after completion of the anastomosis, both in right and left colectomies.

**Results:**

A total of 107 patients undergoing colorectal laparoscopic resections were enrolled: 40 right colectomies, 10 splenic flexure segmental resections, 35 left colectomies, and 22 anterior resections. In 90 % of cases, the indication for surgery was cancer and high ligation of vessels was performed. Based on the fluorescence intensity, the surgical team judged the distal part of the proximal bowel to be anastomosed insufficiently perfused in 4/107 patients (two anterior, one sigmoid and one segmental splenic flexure resections for cancer), and consequently, further proximal “re-resection” up to a “fluorescent” portion was performed. None of these patients had a clinical leak. The overall morbidity rate was 30 %; one patient undergoing right colectomy had an anastomotic leakage, apparently unrelated to ischemia; there were no clinical evident anastomotic leakages in colorectal resections including all low anterior resections.

**Conclusions:**

ICG-enhanced fluorescent angiography provides useful intraoperative information about the vascular perfusion during colorectal surgery and may lead to change the site of resection and/or anastomosis, possibly affecting the anastomotic leak rate. Larger further randomized prospective trials are needed to validate this new technique.

Anastomotic leakage is one of the most dreaded postoperative complications in colorectal surgery. The reported leak rate ranges from 1 to 30 % [1–3] and increases as the anastomosis is more distal [3]. The reported rate of patients with an anastomotic leak that requires surgical revision ranges from 10 to 35 % [4, 5] with a mortality rate ranging from 6 to 22 % [1, 6].

Although several factors have been identified as possible causes of anastomotic leakage (i.e., surgical techniques, patient risk factors, suture material or devices), the complete pathogenesis is still unclear [6, 7].

Poor local tissue oxygenation secondary to inadequate anastomotic vascular perfusion seems to play a key role in the determination of anastomotic viability [8, 9]. Notwithstanding, to date, the most widely used “technique” to evaluate tissue perfusion is surgeon intraoperative visual judgment based on clinical findings, such as color, bleeding edges of resected margins, pulsation and temperature. However, at least two studies have suggested that the clinical judgment of the operating surgeon underestimated the risk of anastomotic leakage in colorectal surgery [10, 11].

Indocyanine green (ICG) is a sterile, anionic, water-soluble but relatively hydrophobic, tricarbocyanine molecule and, once injected into the vascular system, via the intravenous route, binds to plasma proteins. Based on the ability of ICG to become fluorescent when excited by near-infrared light [12], real-time intraoperative organ perfusion evaluation with ICG has been used in several clinical applications [13] including plastic, gastrointestinal and transplant surgery [14–16], as well as bowel perfusion prior to or after colorectal anastomosis [17].

The aim of this study is to analyze our experience with ICG-enhanced fluorescence to evaluate the perfusion of the bowel during laparoscopic colorectal resection.

## Methods

ICG-enhanced fluorescence was performed in all patients undergoing laparoscopic colorectal surgery between May 2013 and October 2014. The study was conducted according to the Declaration of Helsinki.

Patients with a history of adverse reaction to ICG and/or iodine, pregnant and/or lactating women were not included.

Preoperative risk factors including age, sex, American Society of Anesthesiologists (ASA) score, obesity, comorbidities, steroid therapy, preoperative radiation and preoperative transfusions were recorded.

Intraoperative ICG-enhanced fluorescence was used to assess colonic perfusion after intestinal resection, prior to and after completion of the anastomosis, both in right and left colectomies.

Recorded operative details included the site and level of the anastomosis, intracorporeal or extracorporeal anastomosis, vessel ligation, splenic flexure mobilization, use of drainage, intraoperative transfusions and diverting ileostomy.

**Fig. 1:**
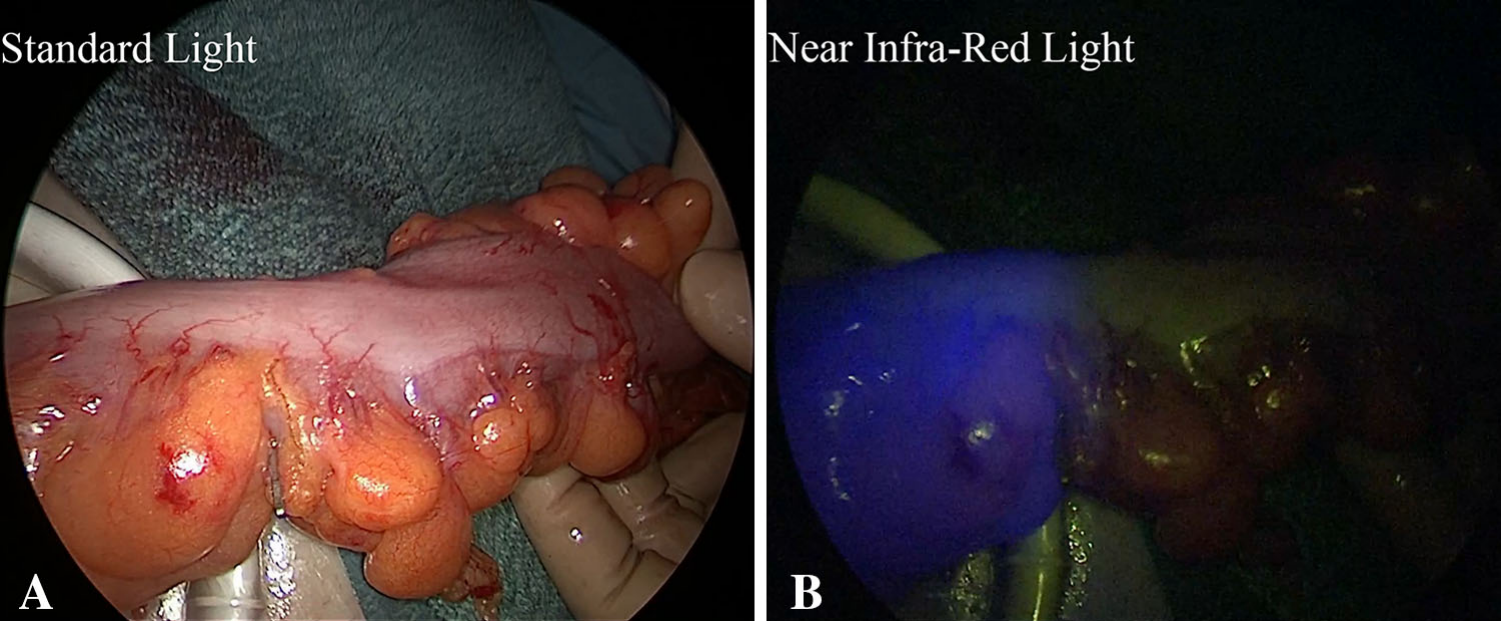
Intraoperative view of the descending colon after division of the mesentery: Surgical clip is placed at the point of planned transection (**A** view with standard light, **B** view with NIR light showing good perfusion)

The site of resection was selected by the surgeon before the ICG injection and marked with a clip (Fig. 1A).

Bowel transection, anastomosis and florescence angiography were performed intra-corporally for right colectomies, while in left colectomies, anterior resection fluorescence angiography and resection were performed outside the abdomen through a mini-laparotomy.

Twenty-five milligrams of ICG was diluted in 10 ml of soluble water, and a bolus of 0.2 mg/Kg was injected intravenously by the anesthesiologist through a peripheral vein after the division of the mesentery and colon, but before anastomosis.

The timing before ICG visualization and intravenous injection was recorded.

Due to the lack of any objective technique to measure the intensity of the fluorescence, perfusion of the bowel was subjectively assessed by the surgical team (main surgeon, main assistant, fellow in minimally surgeon and the assisting resident) who also indicated the perfusion level as “adequate” (meaning uniform to that of proximal colon) or “insufficient.” If poor perfusion was demonstrated, we waited further 180 s prior to re-resect the bowel.

Perfusion images were recorded and assessed in real time (Fig. 1).

Any information or change in timing and/or quantity of injection as well as any change in the transection line after ICG-enhanced fluorescence injection was recorded.

After completion of the anastomosis, another bolus of 0.2 mg/Kg of ICG was injected and a second evaluation of perfusion was made.

An air-leak test was performed after completion of anastomosis at the end of the procedure. The integrity of doughnuts in left hemicolectomies was checked after completion of the anastomosis.

Severity of complications was evaluated according to the Clavien/Dindo classification [18].

## Laparoscopic equipment

The laparoscopic SPIES system (KARL STORZ GmbH & Co. KG, Tuttlingen, Germany) and high-end full high-definition camera system (IMAGE 1 SPIESTM, KARL STORZ) were used in all cases.

We used a xenon light source (D-LIGHT P SCB, KARL STORZ) providing both visible and NIR excitation light. Switching from standard light to NIR was controlled by the surgeon by means of a pedal. ICG fluorescence images appeared blue under NIR excitation, while all other tissues appeared black.

## Results

During the study period, ICG-enhanced fluorescence was used to assess bowel perfusion in 107 patients (mean age 69 ± 12 years, 47 females and 60 males) undergoing right colectomy (*n* = 40), splenic flexure segmental resections (*n* = 10), left colectomy (*n* = 35) and anterior resection (*n* = 22) (Tables 1, 2). Splenic flexure segmental resections are intended for large adenoma not suitable for endoscopic resection or small cancer. In these cases, the inferior mesenteric artery was fully dissected, and the left colic artery ligated at its origin. Similarly, the middle colic artery was dissected, and the left branch ligated at its origin. This is routinely done in our practice since no evidence of oncological benefits of extended resection is reported in the literature.

**Table 1:** Patient characteristics

Number	107
Gender (M/F)	60/47
Average age (years) ± standard deviation	69 (±12)
Indication for surgery	
Colorectal cancer	97
Colorectal adenoma	4
Diverticular disease	4
Crohn’s disease	2

**Table 2:** Patient comorbidities (*n* = 107)

Comorbidity	*N*	%
BMI (kg/m^2^)	25 (±8)	
Cardiovascular	60	56
Respiratory	12	11
Diabetes	17	16
Urogenital	10	9
Neurological	8	7
Hematologic	4	4
Smoker	33	31
Alcohol abuser	3	3

Resection for malignancy was the main indication for surgery (97/107 cases, 90.6 %), and in those cases, a “medial-to-lateral” approach with high ligation of the main feeding vessels was used. Stapled anastomosis was intra-corporeal in 90/107 cases (84.1 %) and extracorporeal in the other 17 patients (15.8 %). Five patients (4.7 %) had undergone preoperative endoscopic stent placement for obstructed malignant cancer as a bridge to surgery.

There were no intraoperative adverse events or conversion to open surgery. Protective ileostomy was carried out in 17 patients (77.3 %) undergoing low anterior resection. (Table 3).

**Table 3:** Perioperative data (*n* = 107)

Operative data	*N*	%
Right colectomies	40	37
Splenic flexure resections	10	9
Left colectomies	35	33
Anterior resection	22	21
<5 cm	7	32
5–8 cm	8	36
>8 cm	7	32
Ostomy	17	16
High ligation vessels*	97	91
Low ligation vessels	10	9
ICG fluorescence imaging detection	107	100
Change in point of transection	4	3.73
Revision of anastomosis	0	0
Operative time (min) (range)	130	(45–180)

* High ligation: ligation of the inferior mesenteric artery proximal to the left colic artery in left colectomies and anterior resections/ligation of collateral branches flush to the superior mesenteric vessels in right colectomies

There were no side effects related to the injection of ICG.

ICG-enhanced fluorescence was detected in 100 % of the cases, and the mean time from IV ICG injection and fluorescence occurred was an average of 57 ± 26 s after injection.

No changes in surgical plan were decided before fluorescence angiography. In all cases, the surgical team judged the perfusion of the colon adequate on standard white light, according to the color of the bowel, and no visible sign of ischemia was evident at visual inspection.

**Fig. 2:**
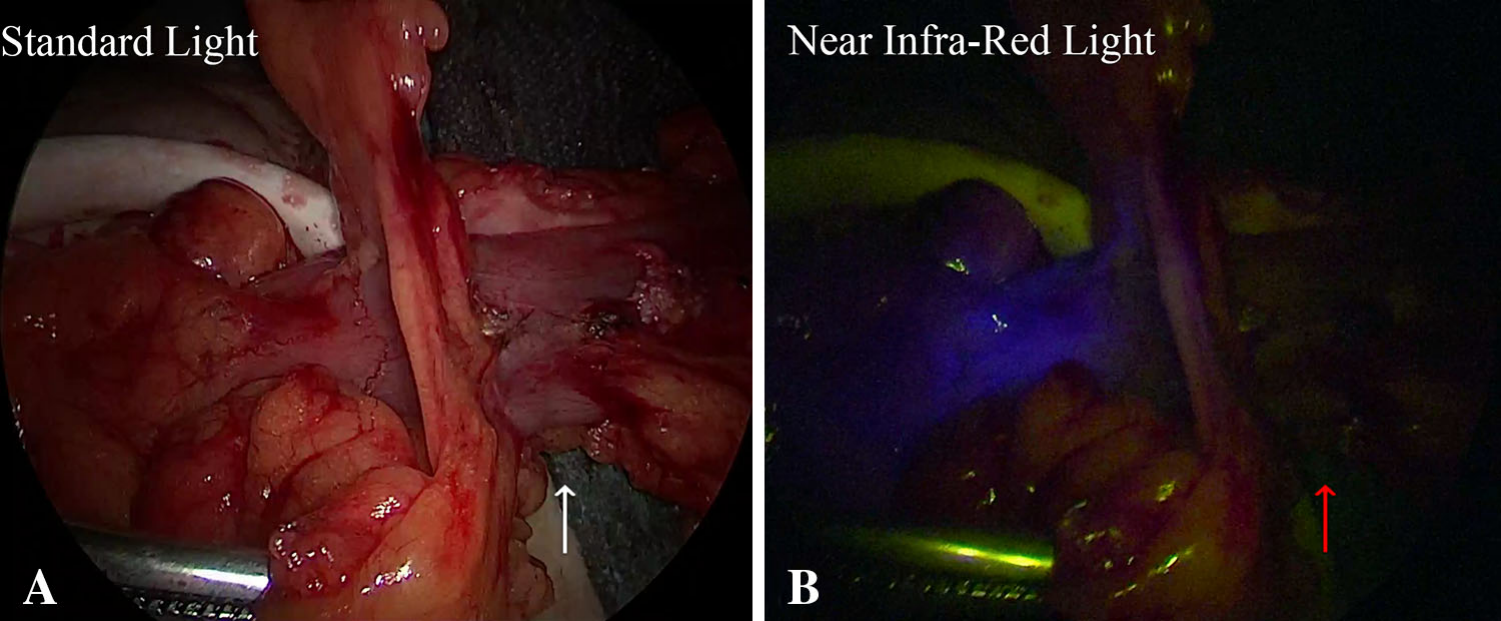
Intraoperative view of the descending colon in a case of hypoperfusion at the point of planned transection (*arrows*)

Based on the fluorescence intensity recorded under NIR light, after injection of ICG, the distal part of the proximal bowel to be anastomosed was judged to be insufficiently perfused and the surgical team opted for further proximal “re-resection” up to an “adequate” fluorescent part in 4/107 patients (3.7 %) (two anterior, one sigmoid and one segmental splenic flexure resections for cancer) (Fig. 2).

The complete lack of fluorescence (3/4 cases) or the macroscopically less fluorescence in comparison with the proximal portion of bowel (1/4 case) was used as criteria to detect the poor perfusion. None of these patients had clinical anastomotic leaks.

In two of these patients, the change in surgical plan entailed further splenic flexure mobilization.

The postoperative course was uneventful in 74/107 (69.1 %) patients, while the remaining 33 (30.8 %) had one or more postoperative complications described in Table 4.

**Table 4:** Postoperative complications (*n* = 107)

Postoperative morbidity	*N*	%
Ileus	6	5.6
Postoperative anemia requiring transfusions	4	3.7
Fever	3	2.8
Pulmonary complications	3	2.8
Wound infections	3	2.8
Urinary tract infections	2	1.8
Incisional hernia	2	1.8
Urinary retention	2	1.8
Rectal bleeding	2	1.8
Postoperative leak	1	0.9
Others	5	4.6
Severity of complication [21]		
Mild (Clavien Dindo 1)	23	21.4
Moderate (Clavien Dindo 2)	9	8.4
Severe (Clavien Dindo 3–5)	1	0.9
Re-operation	1	0.9
Mortality	0	0

One patient underwent re-operation for anastomotic leakage after right colectomy due to incomplete closure of the enterotomy used for stapler introduction. We reported no clinical evident anastomotic leakage in any of the left sided colectomies, including all the low anterior resections.

Reversal of ostomy was performed in 20/22 patients, the anastomotic evaluation prior to reversal was always made either by means of rigid endoscopy (17/20 patients) or gastrografin enema (3/20), and no sign of leaks or stenosis was reported.

## Discussion

Anastomotic breakdown can be due to mechanically faulty (technical or firing/cutting mishaps) or perfusion problems. The former can be sought intraoperatively by testing for air-tightness with air or methylene blue, or intraoperative endoscopy [19], and the second requires other techniques.

Intraoperative assessment of intestinal perfusion and viability usually relies on subjective judgment based on visual inspection. However, it has been shown that surgeon’s subjective evaluation and prediction of anastomotic leakage are poor [11]. In the study by Karliczek et al. [10], surgeons were asked to estimate the risk of anastomotic leakage in 191 patients undergoing colorectal resection with anastomosis; the risk was compared to the actual occurrence of anastomotic leakage postoperatively (26/191 = 13.6 %). The predictive value of surgeons’ opinion was low; the 14 % a priori risk of anastomotic leakage resulted in a low (11 %) posttest odds of correct prediction of anastomotic leakage.

Intraoperative perfusion assessment techniques such as transabdominal Doppler ultrasound [20], transabdominal laser Doppler flowmetry [21] and oxygen spectroscopy [22] have not been widely accepted because such techniques cannot be easily applied in routine clinical practice or have not proven reliable. Vignali et al. [23] measured the microperfusion in 55 patients undergoing colorectal anastomosis with Doppler flowmetry; they changed the anastomotic site when the oxygen content was <50 % from baseline. Notwithstanding, they still observed a 14.5 % leakage rate.

Indocyanine green angiography is a more recent development in colorectal surgery. Kudszus et al. [24] demonstrated a reduction in risk of surgical revision of 60 % (7.5–3.5 %) using fluorescence angiography in a case-matched retrospective study. These data have been confirmed by Jafari et al. [25] during robotic-assisted laparoscopic rectal surgery.

A multicenter prospective trial on fluorescence angiography in colorectal surgery in 147 patients who underwent laparoscopic or robotic left colectomy or anterior resection showed that ICG angiography led to change the surgical plan in eleven patients (7.9 %) [18]; their overall anastomotic leak rate was low (1.4 %, two patients) compared to the literature [26], perhaps because none of the 11 patients with problematic vascular supply detected by this method had an anastomotic leakage after revision.

A recent retrospective case-matched study by Kin et al. [27] on colorectal resection demonstrated that once fluorescence angiography was performed, surgeons decided to change the proximal resection margin in 8/173 patients (4.6 %) but this was not correlated with a significant reduction in the leak rate once compared to the matched group where fluorescence angiography was not performed. Nevertheless, the authors acknowledged several limitations of their study including the retrospective nature, the different period of time where the two groups were compared, the small sample size and the presence of potential confounders between the two groups.

Among the different risk factors for anastomotic leakage, adequate perfusion is a well-recognized prerequisite for complete healing of anastomosis [23, 27, 28].

Adequate vascular supply of the left colon depends on the patency of the inferior mesenteric artery, the left colic artery but also relies on patency of the middle colic artery and the marginal Drumond and eventually Riolan arcades [29, 30]. Anatomic variations are frequent, and aberrations such as absence of the middle colic artery or inadequate vascularization of the splenic flexure are frequent (up to 25 %) [31–33]. High ligation of the inferior mesenteric artery has been documented as a risk factor for anastomotic leakage [34].

The concept of high ligation of the vessels to achieve optimal oncological results [29] suppresses the vascular supply from the left colic artery, and vascularization of the proximal colon is dependent on marginal vessels running off the middle colic artery. With the progressive increase in the aging population undergoing surgery for colorectal disease, radiation, vascular disease can also be a factor of insufficient vascular supply, even in the case of low ligation of the inferior mesenteric artery.

In our study, 92 % patients had high ligation of the inferior mesenteric artery (53 of 57 left colectomies) and 54 % had splenic flexure mobilization (31/57). In the study by Hellan et al. [35], all patients undergoing resection for malignant disease had high ligation. The number of patients undergoing splenic flexure was not stated. Fluorescence evaluation, in contrast to the surgeons’ clinical assessment, led to a more proximal level of division in 16/40 (40 %) of patients. Two leaks occurred in spite of change in anastomotic site in their series [35]. Possible explanations may be the non-standardization of injection and reinjection schedules. Moreover, the authors did not test the anastomoses for air-tightness, not give any details on the vascularization after changing the site of resection, but it is also possible that they were not related to a vascular problem, or there was an undetected discontinuation of the marginal artery.

Patients who have undergone radiation therapy [36] and aortic aneurysm repair [37] might also be at high risk of precarious vascular supply to the splenic flexure [38]. Another risk factor for AL is patients undergoing ultra-low anastomosis. None of the patients undergoing anastomosis <5 cm from the anal verge in our study sustained an AL. This is in accordance with the outcomes of the studies by Kudszus et al. [24] and Jafari et al. [25], where the proportion of patients with low rectal anastomosis and preoperative radiation therapy was high.

Images were obtained in 100 % of our patients, similar to what was obtained for 150 graft perfusions in esophageal surgery reported recently by Zehetner et al. [16] and Hellan et al. [35] for colorectal surgery, but slightly superior to 98.6 % in the PILLAR II study [17].

The technique of intraoperative evaluation still has several unknowns or drawbacks.

One unknown is the optimal dose and timing of ICG injection before assessment. While the ideal time lag between injection and visualization of bile and bile ducts is under scrutiny [38], the wash-out time of the vascular system is much quicker and the precise injection schedule remains to be found. Hellan et al. [35] found that while the microperfusion was usually evident after a few seconds, a bolus injection of ICG was needed to complete the assessment in 7/40 (17.5 %) of patients. The effect of repeated injections of ICG is not known and has not been investigated [39].

Another drawback is that there is still no strict analytic measure to objectively quantify the signal intensity, and the evaluation of images still depends on the surgeon’s judgment. One recent experimental study has targeted this aspect [40] assessing the perfusion of porcine colonic segments after creating a survival ischemic model by ligation and then evaluating the intensity of the fluorescent image. However, this study has several shortcomings. The model of evaluation was based on time to peak intensity. The authors do not provide the rationale for the binary character of good or bad perfusion (over 50 % or <50 % rise in time to peak intensity) on which their model was based. Whether this variable has any bearing on the adequateness of vascular supply to the intestine to prevent anastomotic leakage remains to be shown.

This leads to another unknown factor, which is that the precise blood flow rate needed to ensure satisfactory healing of the intestinal tissue remains unclear at the present time. At least two studies [21, 22] focused on the blood supply assessed using Doppler flowmetry, but although both demonstrated reduced blood flow after resection, it is not clear what is the minimal blood flow to avoid anastomotic leakage [7].

Other variables that have to be considered are the variations in intensity relative to respiratory movements, which measured the intensity at the end of a 20-s apnea period, the distance between the camera and the intestines, the influence of the speed (number of frames/s) of the camera, the quality of the cable for transmission of the light, the body mass index (or any other quantitative measurement of obesity) on the intensity of the image, the amount of ambient white light when the measurement is made outside the abdomen. It also seems important to highlight that perfusion should be tested with the distal segment of intestines (colon) in its definitive position after anastomosis (to be sure that the perfusion does not change due to any tension, or anatomic factor occurring after division and anastomosis.

Notwithstanding, and despite the limitations due to a small-size study, ICG-enhanced fluorescence, as used herein, seems to be safe, reproducible, simple and cost-effective to assess colonic and anastomotic perfusion.

## Conclusions

In our experience, injection of a few milliliters of ICG provides real-time evidence of perfusion of the bowel prior to proximal transection, after division of the mesentery and before the completion of the anastomosis in its definitive anatomic position. If our results will be confirmed by larger prospective studies, intraoperative ICG fluorescent angiography, as described herein, might offer the possibility to lower the rate of anastomotic leaks and the resulting morbidity and mortality rate.
